# Laryngeal polyp associated with reflux disease: a case report

**DOI:** 10.1186/s13256-019-2324-0

**Published:** 2020-01-04

**Authors:** Wael Abdo Hassan

**Affiliations:** 1 Department of Pathology, Faculty of Medicine, Suez Canal University, Ismailia, Egypt; 2 Department of Basic Sciences, Sulaiman Al Rajhi Colleges, PO Box 777, Al Bukayriyah, 51941 Kingdom of Saudi Arabia

**Keywords:** Laryngeal polyp, Gastroesophageal reflux disease, Pathological changes

## Abstract

**Background:**

Among the most common benign laryngeal lesions are vocal nodules and polyps. Their etiology is related to vocal abuse. Gastroesophageal reflux disease is a common condition presenting with a broad spectrum of symptoms, among which are extraesophageal manifestations such as laryngeal polyps.

**Case presentation:**

A 24-year-old Middle Eastern woman presented to the author’s institution with dysphonia and dyspepsia. She underwent endoscopy and was diagnosed with severe reflux disease. In addition, laryngoscopy revealed a polyp at the left vocal cord, and the patient underwent polypectomy. Histopathological examination revealed a laryngeal polyp of telangiectatic type characterized by hyperplastic epithelial covering with reactive atypia, prominent superficial acanthosis with neutrophils, and prominent chronic inflammation and thrombosed vessels in the stroma.

**Conclusion:**

This report focuses on the pathological findings associated with a laryngeal polyp in a young patient diagnosed with severe reflux disease. Acknowledging such characteristic changes in a laryngeal polyp could aid in the diagnosis of gastroesophageal reflux disease.

## Background

Laryngeal polyp is defined pathologically as a noninflammatory response to laryngeal injury usually caused by vocal cord abuse and irritation [1]. “Vocal abuse” refers to misuse of vocal behaviors leading eventually to trauma of the laryngeal mucosa, such as by excessive talking, prolonged and excessive loudness, and the use of inappropriate pitch [2]. Thus, it is more common in singers [1]. The most common clinical manifestation is voice change: generalized and persistent hoarseness, change in voice quality, and increased effort in producing the voice [3]. The usual location of polyps on the superior surface of the cord makes it easy to visualize them by video stroboscopic examination [3]. There are several other causes of laryngeal polyps, despite being less common, such as gastroesophageal reflux disease (GERD), and chronic inhalation of irritants (such as industrial fumes and cigarette smoke). GERD symptoms are described as typical if they present with digestive symptoms (e.g., pyrosis, regurgitation, back breastbone pain) and atypical if the symptoms are extraesophageal, related to the larynx, the pharynx, or other respiratory airways (e.g., cough, dysphonia, dysphagia) [4, 5]. The causes of GERD are multifactorial and include many well-described factors [6], all of which are associated with transient lower esophageal sphincter relaxation that allows a bolus of refluxate to move from the stomach into the esophagus [6]. One of the defense mechanisms in combating gastric reflux is the upper esophageal sphincter (cricopharyngeus), whose pressure might fail to increase in response to esophageal acid exposure, leading to laryngopharyngeal reflux (LPR) [7]. The exact etiology behind the failure of the upper sphincter is uncertain; however, it is also clear that such failure is reversible with effective antireflux treatment [7]. LPR has been associated with vocal cord polyps, vocal cord granulomas, laryngospasm, laryngeal carcinoma, and subglottic stenosis [8]. We report a case of vocal cord polyp diagnosed in a patient with severe GERD.

## Case presentation

A 24-year-old Middle Eastern woman presented to the author’s ear, nose, and throat (ENT) clinic with a complaint of dysphonia that had been present for 2 months. The patient gave no history of any intubations, trauma, or voice abuse. She had associated dyspeptic symptoms. On examination of the ear, nose, and throat, a polypoid lesion was present on the middle one-third region of the left vocal cord measuring approximately 4 × 3 cm in diameter. The patient was referred to the gastroenterology clinic to undergo endoscopy. She was diagnosed with grade C GERD on the basis of the Los Angeles classification system of GERD. Returning to the ENT clinic, the patient was scheduled for surgery for removal of the laryngeal polyp. With the patient under general anesthesia, the polypoid lesion was excised completely by suspension direct laryngoscopy, with no residual tissue left behind.

The excised polyp was fixed in 10% formalin and embedded in paraffin. Serial sections, 5 μm thick, were processed for hematoxylin and eosin (H&E) staining. Immunohistochemistry (IHC) staining was carried out with the streptavidin-biotin method. Primary antibodies (Additional file 1: Table S1) were purchased from Genemed Biotechnologies (San Francisco, CA, USA).

**Fig. 1:**
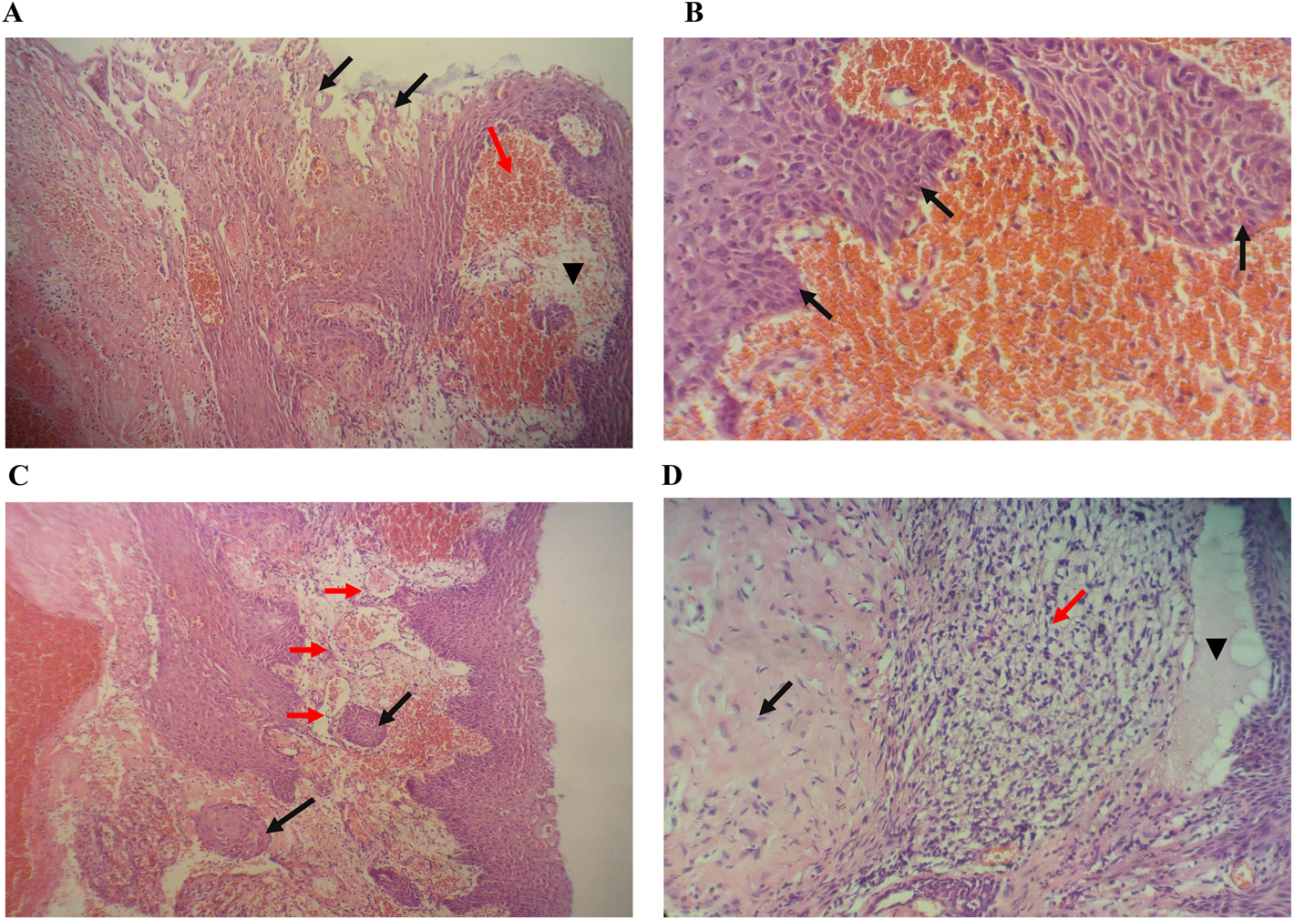
Histopathological features of laryngeal polyp. **a** The polyp is lined with thickened, nonkeratinized, stratified squamous epithelial covering with disruption of superficial layers of epithelium causing intraepidermal microabscesses (*black arrows*). The underlying stroma shows edema (*arrowhead*) and hemorrhage (*red arrow*) (Hematoxylin and eosin stain (H&E) stain, original magnification × 100). **b** Focal elongation of rete ridges is present with mild atypical epithelial cell changes (*black arrows*) (Hematoxylin and eosin stain (H&E) stain, original magnification × 200). **c** The underlying stroma shows numerous proliferated blood vessels (*red arrows*); some are congested. Two nests of uniform squamous epithelium are present with intracellular keratinization and surrounded with basement membrane, representing uniform foci of pseudoepitheliomatous hyperplasia (*black arrows*) (Hematoxylin and eosin stain (H&E) stain, original magnification × 40). **d** The stroma shows dense fibrous tissue deposition (*black arrow*) with numerous focal accumulations of foamy macrophages (*red arrow*). Focal separation of the overlying epithelial covering with edema is present (*arrowhead*) (Hematoxylin and eosin stain (H&E) stain, original magnification × 200)

On gross pathological examination, the specimen consisted of a polypoid mass measuring 4 × 3 cm with a smooth outer surface and a congested cut section. Histologically, as observed by H&E) staining, the polyp was lined with thickened, nonkeratinized, stratified, squamous epithelial covering with disruption of superficial layers of epithelium causing small blisters with intraepidermal microabscesses and underlying areas of hemorrhage (Fig. 1a). The basal epithelial cells were thickened with elongation of rete ridges and mild atypical changes involving the lower third of the epithelial covering in the form of enlarged hyperchromatic nuclei and mild disturbed polarity (Fig. 1b). The underlying stroma showed numerous proliferated blood vessels (Fig. 1c), with some of them dilated with congestion and others with thrombi. Nests of uniform stratified squamous epithelium were present within the stroma and surrounded with basement membrane, representing pseudoepitheliomatous hyperplasia. Dense fibrous tissue deposition was present in the stroma, with focal areas of edema and chronic inflammatory cell infiltrate and numerous focal accumulations of clear cells with enlarged nuclei. Focal separation of overlying epithelial covering by edema was seen (Fig. 1d). Focal ulceration with underlying granulation tissue-like reaction was also observed.

By IHC staining, a negative reaction to p53 in the overlying epithelium was detected, thus excluding the possibility of epithelial dysplasia. In addition, E-cadherin expression was decreased in the superficial layers of the squamous epithelial covering, leading to intercellular spongiosis and the accumulation of neutrophils with microabscess formation. The accumulated clear cells stained positive for CD68 and negative for pan-cytokeratin, confirming their nature as foamy macrophages.

## Discussion and conclusions

Vocal cord polyps and nodules are among the most common prevalent laryngeal lesions (1.0–1.7%), with a slight female and pediatric predominance [9]. They are typically caused by vocal overuse, whereas other irritants may contribute to the development of polyps, such as GERD, smoking, and aspiration of chemical substances [9]. Differentiation between vocal cord nodules and polyps can be done on the basis of both laryngoscopic and pathological parameters [10]. From a histological point of view, vocal cord polyps are classified as telangiectatic and gelatinous polyps. Table 1 summarizes the laryngoscopic and pathological features of vocal cord nodules and polyps [10, 11]. On the basis of these data, the present case represents a vocal cord telangiectatic polyp. GERD has been implicated in the pathogenesis of vocal cord nodules and polyps, possibly due to adductory collision of the vocal cord by the effect of reflux, which results in local trauma, inflammation, and irritation [12]. Moreover, higher presence of pepsin was reported in patients with vocal cord polyps than in a control group [9]. Other laryngeal complications of GERD include paradoxical vocal fold motion, laryngospasm, laryngeal granuloma, stenosis, strictures, ulcers, chronic laryngitis, and laryngeal carcinoma [6, 13–16]. It has been estimated that 55–79% of patients who present with unresponsive hoarseness resulting from chronic laryngitis have acid reflux [14].

**Table 1:** Summary for laryngoscopic and pathological features for laryngeal nodule and polyps

	Vocal cord nodule	Vocal cord polyp
Laryngoscopic findings	Sessile, gray white, usually bilateral, in the anterior or middle third of the vocal folds	Pedicled, gray white/red, usually unilateral, located in the anterior and middle thirds of the vocal folds
Size	Usually less than 0.3 cm	Usually greater than 0.3 cm
Pathological features	Parakeratotic, stratified, squamous epithelial covering overlying dense fibrotic stroma There is prominent basement membrane thickening. Hemorrhage or hemosiderin-laden macrophages are not usually seen.	Two types: 1. Telangiectatic polyps: orthokeratotic, stratified, squamous epithelial covering overlying numerous thin-walled, dilated vessels in edematous stroma; areas of hemorrhage and hemosiderin-laden macrophages are usually seen 2. Gelatinous polyps: stratified, squamous epithelial covering overlying edematous stroma containing fibrin, proliferating fibroblasts, and few thin-walled vessels

The present patient had the clinical and pathological features of a vocal cord polyp associated with GERD. The clinical features included dysphonia associated with dyspepsia. The pathological features included epithelial changes; thickening of the epithelial lining with microabscesses, reactive atypia, pseudoepitheliomatous hyperplasia, and stromal changes; vascular proliferation; hemorrhage; thrombosis edema; fibrous deposition and increased number of foamy macrophages. Despite the presence of atypical epithelial cell changes, a negative reaction to p53 excluded the possibility of dysplasia [17, 18]. On the one hand, acknowledging such a spectrum of changes in a laryngeal polyp could aid in the diagnosis of associated GERD. On the other hand, smoking-associated vocal cord polyps are characterized by significant epithelial hyperplasia, leukoplakia, thickened basement membrane, hyaline degeneration, and lamina propria edema [9, 11].

The preferred treatment in all patients with larynx-associated GERD features is aggressive antireflux therapy combined with speech therapy [14]. Marked improvement could be achieved in the associated laryngeal lesions, such as granulomas and subglottic stenosis, with antireflux therapy alone [15]. Patients with laryngeal polyps, as in the present patient, require surgical intervention for removal of the polyp to avoid airway obstruction and should be given aggressive antireflux therapy preoperatively and maintained on it postoperatively [13].

Regarding the present patient, following laryngeal polypectomy, she was treated with both a proton pump inhibitor (1 mg/kg body mass) and histamine H2 receptor antagonists (1 mg/kg body mass) for 6 months in addition to erythromycin (500 mg twice daily) for 5 days, and an antireflux diet was prescribed by the gastroenterologist. After treatment, the patient was assessed, and she showed dramatic improvement. The patient remains under constant follow-up in a gastrological and laryngological outpatient clinic.

## Supplementary information

**Additional file 1: Table S1.** Antibodies for immunohistochemistry. Lot and working dilutions of antibodies are indicated.

## Data Availability

Data sharing is not applicable to this article, as no datasets were generated or analyzed during the current study.
