# Supplementary material for: Laryngeal polyp associated with reflux disease: a case report
Source: J Med Case Rep. 2020 Jan 4;14:2. doi: 10.1186/s13256-019-2324-0 (PMC6942414; doi:10.1186/s13256-019-2324-0)
Supplement: Supplementary file 1 — Additional file 1: Table S1. Antibodies for immunohistochemistry. Lot and working dilutions of antibodies are indicated. [file 13256_2019_2324_MOESM1_ESM.docx]

**Additional file 1: Table S1 Antibodies for immunohistochemistry. Lot and working dilutions of antibodies are indicated.**

| **Primary antibodies** | **Lot** | **Working dilution** |
| --- | --- | --- |
| P53 | BP-53-12 | 1:100 |
| E cadherin | GM016 | 1:100 |
| CD68 | GR021 | 1:50 |
| Pancytokeratin | 61-0022 | 1:100 |
